# Association between bacterial vaginosis, *Chlamydia trachomatis* infection and tubal factor infertility in Bukavu, Democratic Republic of Congo

**DOI:** 10.1186/s12879-024-09379-w

**Published:** 2024-05-10

**Authors:** Jules Mongane, Erick Hendwa, Dieudonné Sengeyi, Etienne Kajibwami, Freddy Kampara, Serge Chentwali, Claude Kalegamire, Isaac Barhishindi, Yvette Kujirakwinja, Jeanne Beija Maningo, Benjamin Kasago, Guy Mulinganya

**Affiliations:** 1 Faculty of Medicine, Université Catholique de Bukavu, Bukavu, Democratic Republic of the Congo; 2 Department of Obstetrics and Gynecology, Hôpital Provincial Général de Référence de Bukavu, Bukavu, Democratic Republic of Congo

**Keywords:** Bacterial vaginosis, *Chlamydia trachomatis*, Tubal factor infertility, Bukavu, Democratic Republic of Congo

## Abstract

**Background:**

Tubal factor infertility (TFI) is common in sub-Saharan Africa and often secondary to pelvic inflammatory disease (PID). Anaerobes associated with bacterial vaginosis (BV) are also found in PIDs widely dominated by *Chlamydia trachomatis (C. trachomatis)*, whose role in TFI is better demonstrated than that of BV.

**Objectives:**

To determine the prevalence of BV and *C. trachomatis* and to investigate the association between BV, *C. trachomatis* and TFI.

**Methods:**

We included 137 patients treated for infertility between January 2020 and November 2021. Cases were defined as women with infertility aged 18-45 years presenting with TFI (*n* = 52), and controls as infertile women in the same age groups without TFI (*n* = 85). Data on social habits, life style and infertility parameters were collected, and we performed screening for BV and *C. trachomatis*. Multiple regression was used to measure associations.

**Results:**

The prevalence of BV and *C. trachomatis* was 42.3% (58/137) and 23.4% (32/137), respectively. BV (61.5% vs 30.6%, *p*<0.001) and *C. trachomatis* (48.1 vs 8.2%, *p*<0.001) were more frequent in cases of TFI.

BV and *C. trachomatis* increased the risk of TFI approximately 4-fold [aOR: 3.77 (1.61-8.83), *p*=0.002] and 14-fold [aOR: 13.77 (4.59-41.27), *p*<0.001], respectively.

**Conclusion:**

BV and *C. trachomatis* infection are strongly associated with TFI in Bukavu. Prevention and screening should be implemented to reduce the risk of TFI.

## Introduction

Infertility, defined as the inability to achieve a clinical pregnancy after 12 months or more of regular unprotected sexual intercourse [1] and concerns between 9% and 30% of couples [2]. Infertility occurs with an alteration in female and male infertility factors, which may be associated with varying degrees [2].

Previous studies have reported that tubal factors are the main cause of infertility in Africa [3, 4], up to 30% of women consult for infertility [5]. This is essentially related to the high prevalence of pelvic inflammatory disease (PID), a major cause of tubal pathologies related to infertility [6]. PID is caused by the ascent of microorganisms from the lower to the upper genital tract. A variety of organisms are implicated in the etiology of PID, including *Chlamydia trachomatis* (*C. trachomatis*), *Neisseria gonorrhoeae* (*N. gonorrhoeae*), *Mycoplasma genitalium*, and the anaerobic and aerobic bacteria commonly associated with bacterial vaginosis (BV) [7].

BV is a dysbiosis characterized by an imbalance in vaginal flora, with an increase in anaerobic bacteria and a simultaneous disappearance of protective *lactobacilli* [8]. It is associated with a heterogeneous group of pathogens rather than a single etiological agent and includes *Gardnerella vaginalis, Atobopium vaginae, M. hominis*, and various species of *Prevotella, Porphyromonas, Mobiluncus, Sneathia, Peptoniphilus, etc.* [9].

Several women with BV are asymptomatic [10, 11]. Although the prevalence of BV varies greatly from one region to another, depending on the population study, there is a general agreement that BV is more common in black and hispanic women, women who smoke, sexually active women compared to virgin women, lesbians and those with vaginal douching [12, 13].

Well known for its association with adverse pregnancy outcomes [14, 15], the involvement of BV in infertility remains controversial, unlike that of *C. trachomatis* infection [16]. Indeed, although some studies have shown an association between BV and PID and hence tubal factor infertility [17–19], other studies have found no such relationship. In addition, some studies have reported a high prevalence of BV among patients with nontubal infertility and unexplained infertility [20, 21]. To the best of our knowledge, there are no data about the association between VB, C. trachomatis infection and infertility in our area, especially in Bukavu.

Therefore, the aim of this study was to determine the prevalence of BV and *C. trachomatis* infection among patients with infertility and to investigate the association between BV, *C. trachomatis* infection and tubal factor infertility.

## Materials and methods

### Study design

This is an unmatched case-control study involving women who consulted HPGRB’s Department of Obstetrics and Gynecology for infertility treatment between January 2020 and November 2021.

### Sample size and statistical power

The minimum sample size was calculated in *OpenEpi* online software using Kelsey’s formula [22]: 83 patients (28 cases and 55 controls) based on a prevalence of BV of 40% among women without tubal obstruction in a study carried out in Rwanda [23].

### Inclusion criteria

Women between 18 and 45 years old and married.

Women who agree to participate in the study and provide complete information.

Women who have undergone investigation of infertility factors [(ovulatory, hormonal, mechanical and andrological (semen analysis)].

Women who had a vaginal swab for screening for BV and *C. trachomatis* infection.

According to assessment of tubes, Cases were defined as women in infertile couple aged 18-45 years with TFI (*n* = 52).

For controls group, eighty five (*n*= 85) infertile women were recruited at the same period with the same inclusion criteria but without TFI.

### Exclusion criteria

Women who received treatment with clindamycin and/or metronidazole within the last 90 days for any indication.

Women with a history of documented pelvic endometriosis, tubal obstructions following one or more abdominal surgeries or genital tuberculosis.

Isolated male infertility.

Infertility following chemical, surgical or radiation castration.

### Data collection and analysis

Data on anthropometric parameters, sociodemographic characteristics, medical, surgical, gynecological and obstetrical history, habits and lifestyle were collected.

Clinical and paraclinical examinations were performed to investigate infertility factors:

Ovulatory and hormonal factors: Apart from age and cycle length, a blood sample was taken between day 2 and day 5 of the cycle to measure FSH (to assess ovarian reserve). At the same time, transvaginal ultrasound was performed to count the number of antral follicles by summing the number of 2 to 9 mm follicles on both ovaries.

Mechanical factors: ultrasound, hysterosalpingography, or laparoscopy with chromotubation combined with diagnostic hysteroscopy.

The andrological factor: semen analysis.

Screening for BV and *C. trachomatis* was performed during the examination: the patient was in the gynaecological position, then a sterile, nonlubricated Cusco speculum was inserted into the vagina. An intracervical swab was taken to test for *C. trachomatis* antigen using the *One Step Chlamydia trachomatis* Antigen Rapid Test (Colloidal Gold) [24]. A second sample was taken from the posterior fornix using a wooden spatula. The sample was immediately spread onto a slide that was fixed and brought in the AVEONS (Angamiza Vizuri Early Onset Neonatal Sepsis) laboratory, where Gram staining was performed. All Gram-stained slides were read according to Nugent’s morphotype analysis [25] for laboratory diagnosis of BV. Five microscopic fields were read at 100x magnification (with immersion oil) for the presence and quantity of *Lactobacillus* (gram-positive rods), *Gardnerella vaginalis/Bacteroides* (gram-variable coccobacilli) and *Mobiluncus* (gram-negative curved rods).

All slides were scored by two independent readers. In case of discrepancy in results, the criteria were discussed to obtain a consensus on the assessment. If there was no consensus, a third experienced person assessed the slide to make the final categorization.

### Operational definitions

The smear is classified as having either healthy vaginal flora (Nugent score: 0-3), intermediate vaginal flora (Nugent score: 4 to 6) or BV (Nugent score: 7 to 10) [26].

Tubal factor infertility (TFI): radiological or laparoscopic signs of tubal obstruction and/or peri-tubal adhesions altering the normal functional anatomy of the tube [1].

Polycystic ovary syndrome (PCOS): according to the 2003 Rotterdam consensus [27], the presence of two of the following three criteria: oligo/anovulation; clinical or biological hyperandrogenism; polycystic ovaries on ultrasound.

Primary infertility: infertility with no history of clinical pregnancy [28].

Secondary infertility: infertility with a history of clinical pregnancy regardless of outcome [28].

Diminished ovarian reserve (DOR): FSH (follicle stimulating hormone)>10 mUI/ml and/or AFC (antral follicle count) <10 small follicles [29].

Regular cycles: cycle of 24-38 days [30].

### Data management and statistical analysis

Data were recorded in the Excel 2016 database and analysed using STATA 14. Data were summarized into frequencies for categorical variables and into mean± standard deviation (SD) for continuous variables with normal distribution.

We determined the prevalence of BV (Nugent score: 7-10) and *C. trachomatis* infection in the study population and in subgroups. This prevalence was reported with a 95% confidence interval (CI).

Association between dependent variable (TFI) and independent variables were determining using the chi-square test (for BV, *C. trachomatis* infection, type of infertility, occupation); and chi-square of linear trend (for education level). Independent variables with *p*<0.10 in the univariate analysis were included in the logistic regression model. In this model, independent variables with a value of *p*<0.05 are significantly associated with TFI.

### Ethics approval and consent to participate

An informed consent was obtained from all subjects in this study. The study was conducted in accordance with the Declaration of Helsinki, and the protocol was approved by the Ethics committee of the Catholic University of Bukavu and the Ministry of Public Health of DRC (reference number 062/CD/DPS/SK/2017) within the framework of the AVEONS project.

## Results

### Sociodemographic and clinical description of the cases and controls

This study included 137 women with a mean age of 32.9± 5.6 years, ranging from 23 to 45 years. Most of them had higher education, were unemployed and had a monthly household income of over $500 (Table 1). Secondary infertility was found in 67.1% (92/137), with the majority lasting more than 2 years (Table 2).

**Table 1:** Sociodemographic characteristics of cases and controls according to BV

**Parameters**	**Total**	**Tubal factor infertility**					
		**Cases (37.9%)**			**Controls (62.1%)**		
	***n*(%)** **137(100)**	**BV+** **32(61.5)**	**BV -** **20(38.5)**	***p***	**BV +** **26(30.6)**	**BV -** **59(69.4)**	***p***
**Age**				0.258*			0.421*
<25 years	6(4.4)	1(33.3)	2(66.7)		0(0)	3(100)	
25-29 years	36(26.3)	9(60)	6(40)		9(42.9)	12(57.1)	
30-34 years	44(32.1)	14(77.8)	4(22.2)		8(30.8)	18(69.2)	
≥35 years	51(37.2)	8(50.0)	8(50.0)		9(25.7)	26(74.3)	
**Menarches**				0.375			0.472
<13 years	58(42.3)	20(66.7)	10(33.3)		10(35.7)	18(64.3)	
≥13 years	79(57.7)	12(54.5)	10(45.5)		16(28.1)	41(71.9)	
**BMI**				0.074			0.224
18-24 kg/m^2^	38(27.7)	8(44.4)	10(55.6)		8(40.0)	12(60.0)	
25-29 kg/m^2^	52(37.9)	8(57.1)	6(42.9)		8(21.1)	30(78.9)	
≥30 kg/m^2^	47(34.4)	16(80)	4(20)		10(38.46)	17(28.8)	
**Education level**				0.013*			0.129
Primary level	8(5.8)	0(0)	4(100)		3(75.0)	1(25.0)	
Secondary level	43(31.4)	14(77.8)	4(22.2)		6(24.0)	19(76.0)	
Higher education	86(62.8)	18(60.0)	12(40.0)		17(30.4)	39(69.7)	
**Occupation**				0.005*			0.249*
No	54(39.4)	20(83.3)	4(16.7)		13(43.3)	17(56.7)	
Schoolgirl/student	4(2.9)	2(100)	0(0)		0(0)	2(100)	
Independent	34(24.8)	4(40.0)	6(60.0)		7(29.2)	17(70.8)	
Employee	45(32.8)	6(37.5)	10(62.5)		6(20.7)	23(79.3)	
**Monthly income**				*0.592			0.966
<500$	20(14.6)	6(60.0)	4(40.0)		3(30.0)	7(70.0)	
≥500$	117(85.4)	26(61.9)	16(38.1)		23(30.7)	52(69.3)	

^*^Fisher’s test, *BMI* body mass index, *BV* bacterial vaginosis

**Table 2:** Clinical, biological and ultrasound characteristics of cases and controls according to BV

**Parameters**	**Total**	**Tubal factor infertility**					
		**Cases** **52(37.9%)**			**Controls** **85(62.1%)**		
	***n*(%)** **137(100)**	**BV+** **32(61.5)**	**BV -** **20(38.5)**	***p***	**BV +** **26(30.6)**	**BV -** **59(69.4)**	***p***
**Type of infertility**				0.111			**0.039**
Primary infertility	45(32.9)	9(47.4)	10(52.6)		12(46.2)	14(53.8)	
Secondary infertility	92(67.1)	23(69.7)	10(30.3)		14(23.7)	45(76.3)	
**Duration of infertility**				0.482			0.458
<24 months	55(40.2)	16(66.7)	8(33.3)		11(35.5)	20(64.5)	
≥24 months	82(59.8)	16(57.1)	12(42.9)		15(27.8)	39(72.2)	
**SI frequency**				0.634*			0.551*
1-2/week	15(19.9)	2(50.0)	2(50.0)		3(27.3)	8(72.7)	
>2/week	122(89.1)	30(62.5)	18(37.5)		23(31.1)	51(68.9)	
**Irregulars cycles**				0.283*			0.386
Yes	28(20.4)	8(80)	2(20)		4(22.2)	14(77.8)	
No	109(79.6)	24(57.2)	18(42.8)		22(32.8)	45(67.2)	
**DOR**				0.071*			0.149
Yes	24(17.5)	6(100)	0(0)		3(16.7)	15(83.3)	
No	113(82.5)	26(56.5)	20(43.5)		23(34.3)	44(65.7)	
**PCOS**				0.374			0.528
Yes	33(24.3)	10(71.4)	4(28.6)		7(36.8)	12(63.2)	
No	103(75.7)	22(57.9)	16(42.1)		19(29.2)	46(70.7)	
**C. *trachomatis***	32(23.4)		25(48.1)		7(8.2)		<0.001

^*^Fisher’s test, *BV* bacterial vaginosis, *SI* sexual intercourse, *DOR* diminished ovarian reserve, *PCOS* polycystic ovary syndrome

In the hormonal profile study, we found 20.4%(28/137) irregular cycles, 17.5% (24/137) DOR and 24.3% (33/137) PCOS. TFI was the most common infertility factor (37.9%) (Table 2). In the FTI group (cases group), BV was more frequent among women with a secondary education level (77.8% vs. 22.2%, *p*=0.013), those with no occupation and students (83.3% vs. 16.7%, *p*=0.005) (Table 1).

BV was less frequent among women with secondary infertility when they had no TFI (controls group) (23.7% vs 76.3%, *p*=0.039) (Table 2).

### Prevalence of BV and *C. trachomatis* infection

The prevalence of BV and *C. trachomatis* infection in the study population was 42.3%(58/137) (95% CI, 33.9%-51.1%) and 23.4%(32/137) (95% CI, 16.6%-31.3%), respectively.

BV was more frequent among patients with TFI (61.5% vs. 30.6%, *p*<0.001). The same applies to *C. trachomatis* infection (48.1 vs 8.2%, *p*<0.001) (Table 2).

### Association of VB, *C. trachomatis* infection and TFI

BV and *C. trachomatis* infection increased the risk of tubal damage by 4-fold [aOR 3.77 (1.61-8.83), *p*=0.002] and 14-fold [aOR 13.77 (4.59-41.27), *p*<0.001], respectively, regardless of each other and regardless of infertility type, education level and occupation (Table 3).

**Table 3:** Association between BV, *C. trachomatis* infection and TFI

**Parameters**	**TFI prevalence (*n*=52)**	**OR (95%CI)**	***p***	**aOR (95%CI)**	***p***
**BV**					
yes	32/58	4(1.89-8.43)	<0.001	3.77(1.61-8.83)	0.002
no	20/79	1			
***C. trachomatis* Infection**					
yes	25/32	14.4(5.00-41.44)	<0.001	13.77(4.59-41.27)	<0.001
no	27/105	1			
**Type of infertility**			0.473		
Primary infertility	19/45	1			
Secondary infertility	33/92	0.76(0.36-1.59)			
**Education level**			0.574*		
Primary level	4/8	1			
Secondary level	18/43	0.72(0.16-3.27)			
Higher education	30/86	0.54(0.16-2.29)			
**Occupation**			0.495		
No	24/54	1			
Schoolgirl/student	2/2	1.25(0.16-9.54)			
Independent	10/34	0.52(0.21-1.29)			
Employee	16/45	0.69(0.31-1.55)			

^*^chi-square test for linear trend, *OR* odds ratio, *aOR* adjusted odds ratio, *95% CI* 95% confidence interval, *BV* bacterial vaginosis

## Discussion

### General and clinical characteristics of study population

Secondary infertility was found in 67.1% of cases, and tubal factors were the most common factor (37.9%).

In sub-Saharan Africa, secondary infertility is more common than primary infertility [31]. The high rate of secondary infertility in sub-Saharan Africa is thought to be related to the high prevalence of pelvic inflammatory disease secondary to sexually transmitted infections (STIs) and medical interventions in unsanitary conditions: during childbirth or abortion, and especially unsafe abortion [28, 32]. In the meta-analysis by Abebe et al., secondary infertility is predominant in sub-Saharan Africa, and its prevalence varies between 31 and 85% [33].

These results were close to those of several African studies, notably in Nigeria (62-71%), Gambia (59%) and Tanzania (63%) [33], and confirm the WHO report, which mentioned that in sub-Saharan Africa, most couples (52%) suffer from secondary infertility. High rates of secondary infertility (40%) are also observed in Latin America, while in Asia, only 23% of infertile couples suffer from secondary infertility [33].

Regarding infertility factors, tubal alterations are predominant. Indeed, according to the meta-analysis by Abebe et al. [33] on primary and secondary infertility in Africa, the main factor of infertility is tubal factors in developed countries, whereas ovulation abnormalities are the main factor [34].

### Prevalence of BV and *C. trachomatis* infection

The prevalence of BV was 42.3%. Table 4 shows the prevalence of BV among women with infertility, as reported in several studies worldwide. Prevalence varies from one study to another. In sub-Saharan Africa, it is relatively high and close to our results when compared with other continents [35–37]. This can be explained by variations in diagnosis (Amsel criteria, Nugent score, Hay Ison criteria, qPCR, 16S rRNA, IS-pro^TM,^ etc. ), differences in clinical profiles and ethnic differences in the study populations. Indeed, studies in the general population report that the prevalence of BV is higher in black women than in Hispanic and Caucasian women [13]. The frequency of practices such as douching, which is associated with BV, may explain this higher prevalence.

**Table 4:** Prevalence of BV among women with infertility

**Country (City)**	**Prevalence**	**Diagnosis**
**Africa**		
Egypt (Ismailia) [35]	36.6% (26/71)	Nugent score
Egypt (Sohag) [36]	45.5% (398/874)	Spiegel criteria
Egypt (Alexandria) [41]	25% (10/40)	Nugent score
Rwanda (Kigali) [37]	52% (158/312)	Nugent score
**D.R. of Congo (Bukavu) (current study)**	**42.3% (58/137)**	**Nugent score**
**Europe**		
Denmark (Copenhagen) [42]	21% (27/130)-28% (36/130)	qPCR-Nugent
Spain (Barcelona) [43]	23.3% (35/150)	qPCR
Spain (Catania) [44]	6.5% (2/31)	16S rRNA
France (Marseille) [45]	9.4% (3/23)	qPCR-Nugent
Greece (Athens) [46]	36.9% (41/111)	Nugent score
Netherlands (Utrecht) [47]	8.6% (17/198)	Nugent score
Netherlands (Maastricht) [48]	17.7% (34/192)	IS-pro^TM^
United Kingdom (Leeds) [49]	24.3% (182/749)	Hay et al
Ireland (Dublin) [50]	10% (12/120)	Nugent score
United Kingdom (London) [51]	18.5% (37/199)	Nugent score
United Kingdom (Leeds) [52]	24.6% (190/771)	Hay et al
United Kingdom (Bristol) [53]	25.6% (77/301)	Hay et al
United Kingdom (Glasgow) [18]	16.3% (40/331)	Amsel criteria and Gram stain
**Asia**		
India (Mumbai) [20]	25.9% (29/112)	Nugent score
Iran (Tehran) [54]	7.3% (29/399)	Nugent score
Japan (Tokyo) [55]	44.3% (44/79)	16S rRNA
Turkey (Ankara) [56]	37.8% (45/120)	Nugent score
**America**		
United States (New York) [21]	4.2% (14/331)	Nugent score
United States (Seattle) [57]	11% (10/91)	Nugent score
United States (Washington) [58]	13.2% (12/91)	Nugent score

*16S rRNA* 16S ribosomal ribonucleic acid (rRNA), *IS-pro*^*TM*^ interspace profiling, *qPCR* quantitative polymerase chain reaction

The prevalence of *C. trachomatis* infection was 23.4% in the study population. Our result was higher than the prevalence of 8.7% and 18.2% found in two African studies using the rapid antigenic test [38, 39]. A recent meta-analysis of *C. trachomatis* infection in North Africa and the Near East reported a prevalence of 12.4% in women with infertility [40].

### Association between BV, *C. trachomatis* and TFI

A high prevalence of BV was found among patients with TFI compared to those without TFI (61.5% vs. 30.6%, *p*<0.001). BV was therefore strongly associated with a 4-fold increased TFI risk, regardless of *C*. *trachomatis* infection.

Very few studies have investigated this association: in three British studies involving patients undergoing in vitro fertilization for infertility, Gaudoin et al. [18]; Liversedge et al. [53]; Wilson JD et al. [49] reported that patients with tubal infertility were 6-, 2- and 3-fold more likely to have BV than those without tubal infertility, with prevalences of 87.5%, 31.5% and 36.4%, respectively.

In Africa, Dhont et al. [37], in a study of predictors of infertility in Rwanda, reported that BV was 2-fold more likely to have tubal infertility, with a prevalence of 25% according to the Amsel criteria and 56% according to the Nugent score. Innoncent Durugbo et al. [4] compared BV between women with tubal infertility and women without infertility in Nigeria and found a higher prevalence (28.1%) among women with tubal infertility.

All these results support the existence of an association between BV and TFI through PID, even though they do not establish a cause-and-effect relationship.

However, in an American study of women undergoing in vitro fertilization, patients with idiopathic infertility were more likely to have BV than women with other causes of infertility; highlighting the potential role of pro-inflammatory cytokines such as IL-1beta and IL-8 at the cervical level in women with altered vaginal flora [21].

Women with TFI had a higher prevalence of *C. trachomatis* than those without tubal alterations (48.1% vs. 8.2%, *p*<0.001). Analyses adjusted for BV showed that *C. trachomatis* infection is strongly associated with tubal alterations (with 14-fold increased risk).

*C*. *trachomatis* infection can induce PID, which can lead to tubal infertility, ectopic pregnancy and chronic pelvic pain [16].

Although *C. trachomatis* is not the only germ incriminated [16], several studies have also found a strong association between *C. trachomatis* infection and tubal damage [59–64].

The study main limitations were as follows:

The sample size does not allow us to generalize our conclusions to the entire population.

 This study does not establish a causal link between BV, *C*. *trachomatis* infection and TFI.

Using the rapid antigenic test may underestimate certain results compared with most studies using serological tests.

Using the Nugent score, we endorse the limit of interpretation of intermediate flora that can be better categorized by genome sequencing and qPCR methods.

In conclusion, despite the limitations, these data highlight the high prevalence of bacterial vaginosis (42.3%) and *C*. *trachomatis* infection (23,4%) among women with infertility and their strong association with tubal factor infertility, which is the major cause of infertility in our environment. Prevention and screening should be implemented to reduce the risk of infertility.

## Data Availability

The datasets used and/or analyzed during the current study are available from the corresponding author on reasonable request.

## Abbreviations:

16S rRNA: 16S ribosomal ribonucleic acid
AFC: Antral follicle count
AVEONS: Angamiza Vizuri Early Onset Neonal Sepsis
BV: Bacterial vaginosis
DOR: Diminished ovarian reserve
FSH: Follicle Stimulating Hormon
HPGRB: Hôpital provincial général de référence de Bukavu
IL: Interleukin
IS-pro^TM^: Interspace profiling
PCOS: Polycystic ovary syndrome
PID: Pelvic inflammatory disease
qPCR: Quantitative polymerase chain reaction
TFI: Tubal factor infertility
UCB: Université Catholique de Bukavu
